# The Interplay of Lifestyle and Adipokines in the Non-Obese Stroke-Prone Spontaneously Hypertensive Rats

**DOI:** 10.3390/antiox12071450

**Published:** 2023-07-19

**Authors:** Renáta Szabó, Denise Börzsei, Alexandra Hoffmann, Viktória Kiss, András Nagy, Szilvia Török, Médea Veszelka, Nikoletta Almási, Csaba Varga

**Affiliations:** Department of Physiology, Anatomy and Neuroscience, Faculty of Science and Informatics, University of Szeged, H-6726 Szeged, Hungary; borzseidenise@gmail.com (D.B.); hoffmannalexandra1228@gmail.com (A.H.); k.viktoria007@gmail.com (V.K.); nagy.andras.levente99@gmail.com (A.N.); szilva77@gmail.com (S.T.); veszmed@gmail.com (M.V.); almasi.niki91@gmail.com (N.A.); vacs@bio.u-szeged.hu (C.V.)

**Keywords:** *SHRSP* rat, adipokine profile, lipid peroxidation, high-fat diet, exercise

## Abstract

Although the morphological features and functions of adipose tissue are well-described in obesity-prone animal models, less information is available on animals such as the *stroke-prone spontaneously hypertensive* (*SHRSP*) strain with cardiovascular abnormalities, which is not characterized by excessive adiposity. Our aim was to focus on lifestyle-induced (type of diet and physical exercise) effects on adipokine profile and lipid peroxidation in *SHRSP* rats. In our study, male *Wistar-kyoto* (control) and *SHRSP* rats were used. *SHRSP* rats were fed either standard chow or a high-fat diet with 40% fat content (HFD). One group of the animals was placed into cages fitted with a running-wheel; thus, the dietary and training period started at the same time and lasted for 12 weeks. At the end of the experimental period, adiponectin, leptin, omentin, and chemerin concentrations were determined from adipose tissue and serum. Besides adipokines, malondialdehyde (MDA) levels were also measured. Twelve weeks of HFD significantly decreased adiponectin and omentin concentrations of both adipose tissue and serum, which were ameliorated by physical exercise. Serum leptin, chemerin, and MDA values were elevated in HFD groups; however, physical exercise was able to mitigate these adverse changes. Our results underpin the crosstalk between lifestyle changes and dysfunctional adipose tissue in *SHRSP* rats.

## 1. Introduction

Dysregulated fat accumulation is a multifactorial condition that originates from genetic factors and various lifestyle habits. Both overconsumption of energy-dense nutrients and lack of physical activity can be major contributors to excessive fat accumulation, which serves as the pathological basis of obesity. Changes in lifestyle and dietary habits lead to morphological (i.e., hypertrophy or hyperplasia) and functional alterations of adipose tissue [1]. Adipose tissue is a dynamic and multicellular organ, which secretes bioactive molecules, such as hormones, adipokines, and cytokines with a broad-range of biological mechanisms that contribute to immunity, inflammation, and matrix remodeling, as well as determine the adipocyte-dependent and systemic metabolic/redox states [2]. There is considerable evidence that the adipocyte-secreted adipokines, namely adiponectin and leptin, play an important role in the energy homeostasis, and additionally, the adiponectin-leptin ratio serves as a functional biomarker of adipose tissue inflammation [3,4]. The implication is that obesity-induced fat accumulation results in altered secretion of adipokines, which can eventually result in a dysregulated adipose tissue. Altered nutritional and low-grade inflammatory statuses are possible triggers of oxidative stress [5]. Oxidative stress can result in a detrimental shift in oxidant/antioxidant homeostasis; thus, the generation of reactive oxygen species (ROS) further exacerbates obesity-dependent conditions. Accelerated ROS accumulation and inflammation, vice versa, can lead to lipid peroxidation and eventually to the dysfunction of adipose tissue. Previously, Zhou et al. summarized that excess calorie intake can be associated with adipose tissue dysfunction characterized by oxidative stress and inflammation [6]. Moreover, they concluded that these pathological processes can contribute to the incidence and progression of cardiovascular comorbidities. Although the obesity-related changes are well-described, there is less information about strains that are characterized by the absence of excessive fat accumulation. Previous studies demonstrated that both *spontaneously hypertensive (SHR*) and *stroke-prone spontaneously hypertensive (SHRSP)* strains are unique models of cardiovascular morbidities such as hypertension [7,8]. However, their phenotypes are protected against adiposity and changes in adipokine pattern, and the effects of lifestyle habits are not fully elucidated in the non-obese *SHRSP* strain, which possesses cardiovascular abnormalities, among others. 

In order to gain insight into these regulatory circuits, we first aim to compare the values of *Wistar-kyoto* (*WKY*) control rats with those of the age-matched *SHRSP* strain. *WKY* rats are normotensive and the closest control for the *SHRSP* strain. Beside this comparison, we set a goal to examine the impact of high-fat diet and physical exercise on the regulatory mechanisms of adipose tissue in the non-obese *SHRSP* rats.

## 2. Materials and Methods

### 2.1. Experimental Protocol and Sample Collection

In our study, five-week-old male *SHRSP* and *WKY*, *n* = 6 rats were housed at a constant temperature of 20–23 °C with a 12 h light-dark cycle according to the Directive 2010/63/EU. 

After two weeks of acclimatization period, *SHRSP* rats were randomized based on type of diet and physical exercise. Based on the diet, rats were divided into CTRL (*n* = 6) or HFD groups (*n* = 8). Rats in CTRL groups served as control animals on a standard diet, whereas HFD rats were fed ad libitum with a high-fat diet containing 40% fat from lard. HFD was prepared by mixing the ground standard rodent diet with 40% commercially available lard. In order to examine the potential effects of physical exercise training, one group of animals was assigned to exercising group (R); therefore, these rats in pairs were placed into cages fitted with a running wheel. The training protocol was defined as a voluntary wheel-running exercise to minimize any potential stress effects. In our specific cages, the running wheel was a part of the rat’s living space to which the animals had free access for 24 h a day. The dietary and the training period started at the same time and lasted for 12 consecutive weeks (*n* = 8 in *SHRSP*/CTRL + R, *n* = 8 in *SHRSP*/HFD + R) [9]. In our experiment, all efforts were made to minimize the number of animals as well as animal suffering.

**Figure 1:**
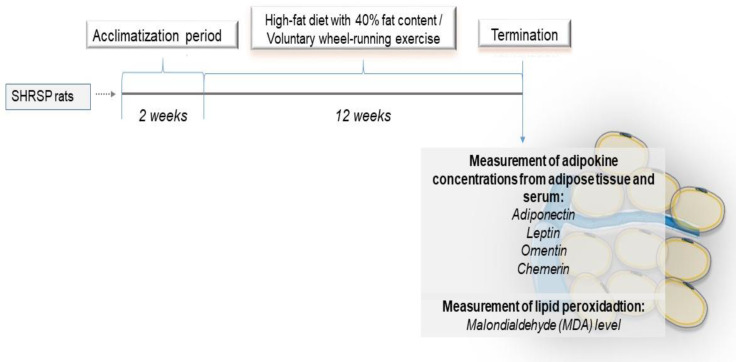
Experimental design of the study.

At the end of the 12-week experimental period, rats were anesthetized and cardiac puncture was carried out to collect blood serum samples. Serum samples were obtained by centrifugation at 2000× *g* for 20 min at 4 °C. Inguinal adipose tissue depots were excised and frozen in liquid nitrogen and finally stored at −80 °C until analysis. The experimental design is illustrated in Figure 1.

### 2.2. Ethical Approval

All procedures were approved by the Institutional Ethics Committee (approval code: XX/2018.) and were performed in accordance with the standards of the European Community guidelines on the care and use of laboratory animals (XX./983/2021).

### 2.3. Determination of Adipokine Profile with ELISA 

At the end of the 12 weeks of the experimental period, leptin, adiponectin, omentin, and chemerin concentrations were measured from both adipose tissue and serum. 

To determine adipokine concentrations in adipose tissue, adipose tissue samples were homogenized (Ultra-Turrax T8, 2 × 30 s) in an ice-cold mixture of 0.2 M monobasic sodium phosphate and 0.2 M dibasic sodium phosphate (phosphate buffer, pH 7.4) and then centrifuged at 2000 rpm for 20 min at 4 °C. For measurement of serum adipokine concentrations, the centrifuged and separated serum samples were used. Both serum and adipose tissue adipokines were assayed with commercial kits purchased from GenAsia. Briefly, blank, standard, and test samples were pipetted to a 96-well ELISA plate. A volume of 10 µL Biotin antibody was added to the test samples and then both standard and test samples were completed with 50 µL streptavidin-HRP. The mixture was incubated at 37 °C for 60 min. After washing procedure, 50 µL chromogen A and then 50 µL chromogen B were pipetted to each well for color development. Finally, the reaction was stopped with 50 µL stop solution and the absorbance was measured at OD 450 nm (Benchmark Microplate reader, Bio-Rad, Hercules, CA, USA). 

Adipokine concentrations measured from adipose tissue were normalized to protein content. Aliquots of 20 μL of the diluted samples were mixed with 980 μL distilled water and 200 μL Bradford reagent. After mixing and following 10 min incubation, the standard and test samples were measured spectrophotometrically at 595 nm. Protein values, leptin, and omentin levels were defined as ng/mg protein, adiponectin concentrations were defined as µg/mg protein, and chemerin levels were expressed as pg/mg protein. For evaluation of serum adipokine concentrations, serum leptin levels were defined as ng/mL, serum adiponectin concentrations were defined as mg/L, and serum omentin and chemerin levels were expressed as ng/L.

### 2.4. Determination of Lipid Peroxidation (MDA)

The ratio of lipid peroxidation was determined by measuring the concentration of malondialdehyde (MDA). Adipose tissue samples were homogenized (Ultra-Turrax T8, 2 × 30 s) in 20 mM Na-phosphate buffer (pH 3 + 0.5% TritonX-100) and then centrifuged at 3000 rpm for 20 min. Subsequently, the oil layer was removed and the clean supernatant was collected in another tube. During assay procedure, either 50 µL standards or 50 µL of test samples was transferred to a 96-well ELISA plate, which was followed by pipetting of 10 µL color reagent solution into both standard and test samples. The mixture was incubated at room temperature for 20 min. In order to induce the reaction phase of the assay, 40 µL of reaction solution was added to the assay volume and the final reaction mixture was incubated at room temperature for 45 min. Optical density was measured with a microplate reader (Thermo Scientific Multiskan Ex, Waltham, MA, USA) at OD 700 nm. MDA concentration was expressed as µM/mL.

### 2.5. Statistical Analyses

Normality of data and homogeneity of variances were verified with the Shapiro-Wilk test. In order to analyze the differences between *WKY*/CTRL and the *SHRSP* groups, one-way ANOVA with Tukey post-testing was used and & *p* ≤ 0.05 was considered as significant. For comparison, two-way ANOVA with Holm-Sidak multiple comparison test was carried out and * *p* ≤ 0.05 as well as # *p* ≤ 0.05 were considered as significant. Statistical analysis was carried out with the use of SigmaPlot 12.0 for Windows (Systat Software Inc., San Jose, CA, USA). 

## 3. Results

### 3.1. Body Weight Changes

Table 1a shows the mean body weights of both *WKY* and *SHRSP* groups measured at the start and at the end of the 12-week experimental protocol. Table 1b indicates the body weight changes, which were calculated from the differences between the individual initial and the individual final body weights of the animals. It is shown that 12-week HFD and training protocol resulted in a significant increase in both *SHRSP*/CTRL + R and *SHRSP*/HFD + R groups compared to the non-running counterparts. Although HFD factor in itself did not cause a significant change in body weight compared to *SHRSP*/CTRL group, the significant analyses show that the running factor has a significant impact on body weight gain. 

### 3.2. Adiponectin Concentrations

**Figure 2:**
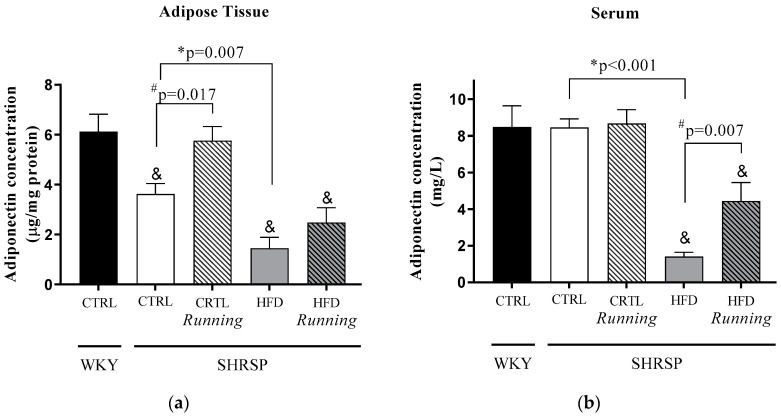
(**a**) Effects of high-fat diet and physical exercise on adiponectin concentrations in adipose tissue (expressed as µg/mg protein). The results are shown as the mean ± S.E.M. *n* = 3–5. (**b**) Effects of high-fat diet and physical exercise on serum adiponectin concentrations (expressed as mg/L). The results are shown as the mean ± S.E.M. *n* = 4–5. & *p* < 0.05: statistical significance compared to the *WKY*/CTRL group, * *p* < 0.05: statistical comparison between *SHRSP*/CTRL and *SHRSP*/HFD groups, # *p* < 0.05: statistical comparison between running and the respective non-running groups, *WKY = Wistar-kyoto* (control) rats, CTRL= standard chow, HFD= high-fat diet, R = running, *SHRSP= stroke-prone spontaneously hypertensive* rats.

Our comparison between the *WKY*/CTRL and *SHRSP* groups shows that HFD resulted in the most significant decrease in adiponectin concentrations in adipose tissue and serum samples. Subsequently, our aim was to assess whether 12 weeks of high-fat diet and physical exercise resulted in any changes in adiponectin concentrations. Our results clearly show that an energy-dense diet with 40% fat content significantly decreased adiponectin concentrations in both adipose tissue and serum samples compared to the *SHRSP*/CTRL animals. In case of adipose tissue, this concentration decrease was ~50%, while the serum adiponectin level was reduced by a quarter compared to *SHRSP*/CTRL animals. Twelve weeks of exercise were able to mitigate these pathological alterations, and reached a statistical significant increase in the adipose tissue of *SHRSP*/CTRL running group and in the serum sample of the *SHRSP*/HFD running group. Data are presented in Figure 2a,b. 

Although both running and HFD factors, in themselves, possess significant impacts on adiponectin concentrations, interactions between these two parameters were not observed. The interaction value is presented in Table 2a,b. 

### 3.3. Leptin Concentrations

**Figure 3:**
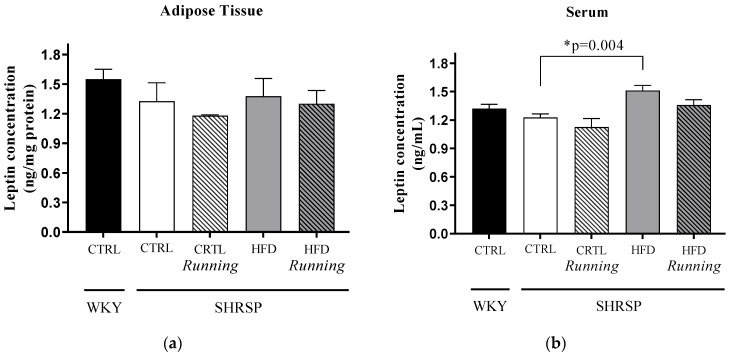
(**a**) Effects of high-fat diet and physical exercise on leptin concentrations in adipose tissue (expressed as ng/mg protein). The results are shown as the mean ± S.E.M. *n* = 3–6. (**b**) Effects of high-fat diet and physical exercise on serum leptin concentrations (expressed as ng/mL). The results are shown as the mean ± S.E.M. *n* = 5–6. * *p* < 0.05: statistical comparison between *SHRSP*/CTRL and *SHRSP*/HFD groups, *WKY* = *Wistar-kyoto* (control) rats, CTRL= standard chow, HFD= high-fat diet, *SHRSP*= *stroke-prone spontaneously hypertensive* rats.

Comparison between *WKY*/CTRL and *SHRSP* rats did not show any significant difference in leptin concentrations. When we analyzed the impact of lifestyle changes on the *SHRSP* groups, we found that 12 weeks of HFD resulted in a significant increase in serum leptin concentration compared to the *SHRSP*/CTRL values. Data are presented in Figure 3a,b. We found no interaction between running and diet factors in case of leptin concentrations (Table 3a,b).

### 3.4. Omentin Concentrations

**Figure 4:**
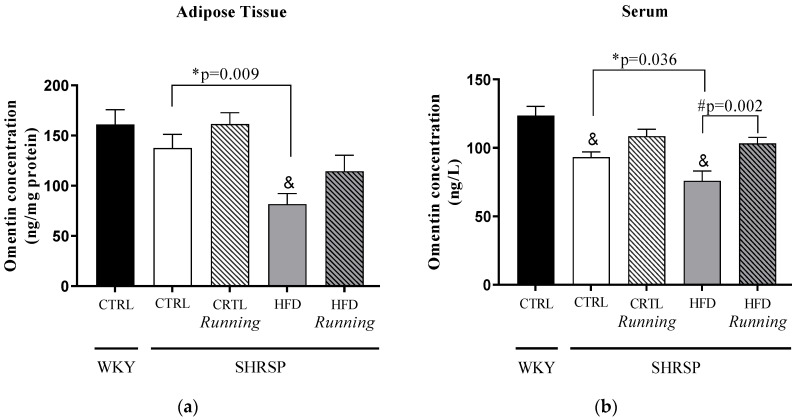
(**a**) Effects of high-fat diet and physical exercise on omentin concentrations in adipose tissue (expressed as ng/mg protein). The results are shown as the mean ± S.E.M. *n* = 3–5. (**b**) Effects of high-fat diet and physical exercise on serum omentin concentrations (expressed as ng/L). The results are shown as the mean ± S.E.M. *n* = 5–6. & *p* < 0.05: statistical significance compared to the *WKY*/CTRL group, * *p* < 0.05: statistical comparison between *SHRSP*/CTRL and *SHRSP*/HFD groups, # *p* < 0.05: statistical comparison between running and the respective non-running groups, *WKY* = *Wistar-kyoto* (control) rats, CTRL= standard chow, HFD = high-fat diet, *SHRSP = stroke-prone spontaneously* hypertensive rats.

As shown in Figure 4a,b, we found a HFD-induced significant decrease in omentin concentration compared to the *WKY*/CTRL group. Similar to the results of adiponectin concentration changes, lifestyle intervention has a significant impact on omentin concentrations. Twelve weeks of high-fat diet resulted in a significant decrease in omentin concentrations of both the adipose tissue and serum of *SHRSP*/HFD rats compared to the *SHRSP*/CTRL group. Our findings also highlight that these HFD-induced adverse changes were ameliorated by physical exercise, which was statistically significant in serum samples. Table 4a,b shows the interaction between running and diet factors; however, we did not found any significant changes.

### 3.5. Chemerin Concentrations

**Figure 5:**
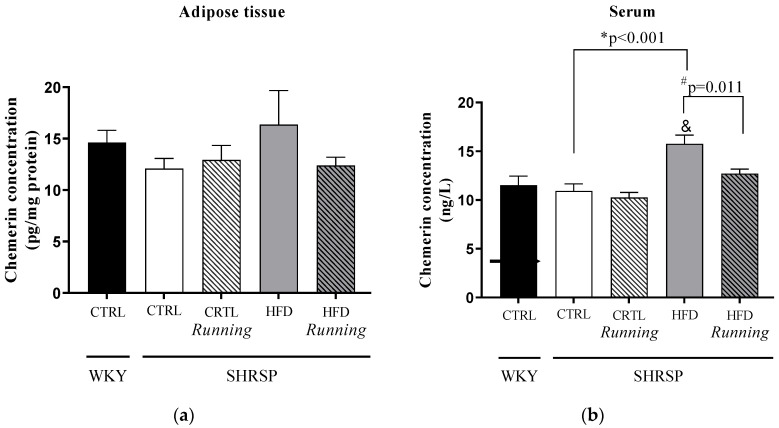
(**a**) Effects of high-fat diet and physical exercise on chemerin concentrations in adipose tissue (expressed as pg/mg protein). The results are shown as the mean ± S.E.M. *n* = 3–6; (**b**) Effects of high-fat diet and physical exercise on serum chemerin concentrations (expressed as ng/L). The results are shown as the mean ± S.E.M. *n* = 4–6. & *p* < 0.05: statistical significance compared to the *WKY*/CTRL group, * *p* < 0.05: statistical comparison between *SHRSP*/CTRL and *SHRSP*/HFD groups, # *p* < 0.05: statistical comparison between running and the respective non running groups, *WKY* = *Wistar-kyoto* (control) rats, CTRL= standard chow, HFD= high-fat diet, *SHRSP* = *stroke-prone spontaneously hypertensive* rats.

As shown in Figure 5b, 12 weeks of HFD with 40% fat content significantly increased the serum chemerin concentration in SHRSSP animals compared to the *WKY* strain. A similar statistical difference was observed between the *SHRSP*/CTRL and *SHRSP*/HFD groups. When we analyzed the effects of voluntary wheel-running, a statistically significant reduction was found in the *SHRSP*/HFD running group compared to the *SHRSP*/HFD animals. Table 5a,b shows the potential interaction between running and diet factors; however, there was no significant effect of these factors on chemerin concentrations.

### 3.6. Determination of Lipid Peroxidation

**Figure 6:**
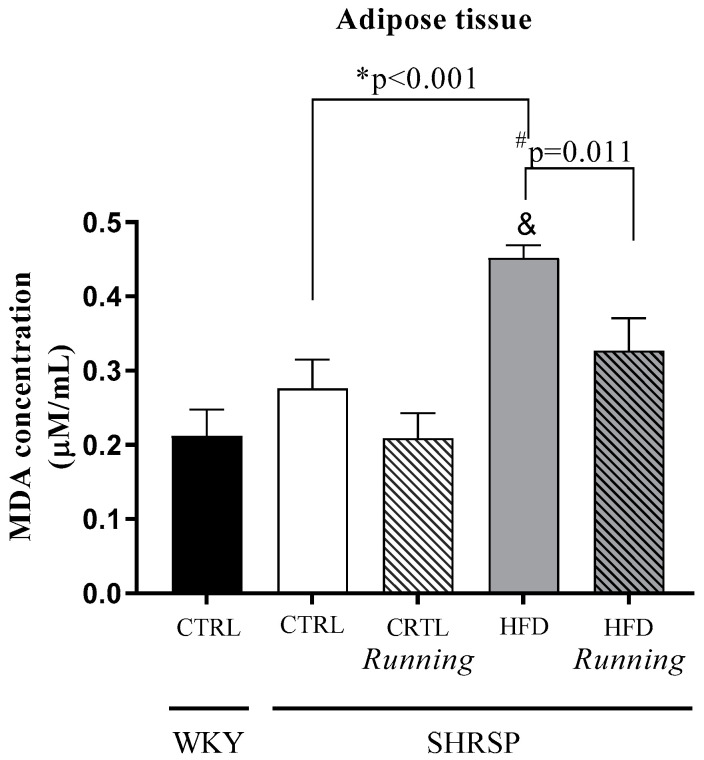
Effects of high-fat diet and physical exercise on lipid peroxidation (MDA, expressed as µM/mL). The results are shown as the mean ± S.E.M. *n* = 4–7; MDA= malondialdehyde. & *p* < 0.05: statistical significance compared to the *WKY*/CTRL group, * *p* < 0.05: statistical comparison between *SHRSP*/CTRL and *SHRSP*/HFD groups, # *p* < 0.05: Statistical comparison between running and the respective non-running groups, *WKY* = *Wistar-kyoto* (control) rats, CTRL= standard chow, HFD= high-fat diet, *SHRSP*= *stroke-prone spontaneously hypertensive* rats.

In order to evaluate the ratio of lipid peroxidation, MDA level was determined from adipose tissue depots. Comparison between *WKY*/CTRL and *SHRSP* groups shows a significant difference in MDA concentration between *WKY*/CTRL and *SHRSP*/HFD groups. The examination of the lifestyle-induced effects shows that 12 weeks of HFD resulted in a significant increase in lipid peroxidation, since the MDA level was significantly increased in the *SHRSP*/HFD group compared to the *SHRSP*/CTRL animals. However, voluntary wheel-running exercise was a potential non-invasive tool to mitigate HFD-induced adverse MDA elevation. Data are presented in Figure 6 and Table 6. 

## 4. Discussion

It is well-known that lifestyle factors such as diet and physical activity have deleterious impacts on many organs and tissues, which can lead to the development and progression of various non-communicable diseases. In these pathological circuits, adipose tissue is also affected; thus, unbalanced lifestyle factors serve as major contributors to the abnormal functionality of adipose tissue, which can consequently manifest in an altered adipokine release and inflammatory processes as well as oxidative damages. Dysfunctional adipose tissue-related adipokine release can have a direct effect on inflammatory and cardiovascular regulatory functions [10]. 

Although a large number of previous studies focus on obesity-related changes and their accompanying metabolic co-morbidities in both human and genetic or diet-induced obese animal models [11,12,13], crucial research and medical questions remain open to underpin the possible target points of therapeutic treatments in this scenario. These observations emphasize the necessity to examine the major features and underlying mechanisms in different clinically relevant animal models. Therefore, the aim of our study was to clarify the interplay of lifestyle habits and adipokines in a special, non-obese genetic *SHRSP* rat strain, which possesses cardiovascular abnormalities. We found that unfavorable nutrition and lack of physical exercise led to a shift in adipokine profile and increased oxidative stress in animals with essential hypertension. Our results also proved that 12 weeks of voluntary physical exercise, as a non-pharmacological strategy, was able to improve the pathological alterations.

For a long time, adipose tissue has been considered as a dynamic storage organ, which can be modified by genetic and environmental factors. With the advent of deep research into this area, it is now recognized that adipose tissue is one of the largest endocrine organs with an impact on a broad-range of physiological processes [14]. It is generally determined that adipose tissue has a unique ability to expand to an almost unlimited extent, and the subsequent morphological changes can occur via either hypertrophia or hyperplasia. In response to overnutrition, adipose tissue undergoes a quality/quantity-based remodeling process and an altered adipokine and proinflammatory cytokine secretion [15]. As a growing body of studies investigates the changes in adipose tissue-derived secretomes in obese animal models [11,12,16], further experimental models and researches are still required to understand the complex regulatory background of lifestyle alterations.

In our present study, we put a spotlight on adipocyte function and examined the consequences of a high-fat diet in adipokine secretion in *SHRSP* rats. Although the blood pressure changes were not monitored in this study, it is well-known that *SHRSP* strain is a unique genetic model of severe hypertension and cerebral stroke, which was selected from *spontaneously hypertensive rats (SHR)* [17]. Previously, we proved that both energy-dense nutrition and lack of physical exercise altered the redox-sensitive parameters. These unfavorable lifestyle factors shifted the antioxidant/oxidant homeostasis towards oxidative conditions as well as exacerbating the inflammatory status. Eventually, these changes made the heart more vulnerable to hypertensive pathological processes [9]. A large quantity of evidence has confirmed a strong correlation between changes in adipose tissue and hypertension [18,19]. Adverse transformation in abundance, distribution, cellular composition, or endocrine signaling can worsen the cardiovascular function and homeostasis. Schütten et al. discussed that dysfunctional adipose tissue can overactivate the renin-angiotensin-aldosterone system (RAAS), and this mechanism plays a crucial role in the incidence or manifestation of hypertension. They also showed that the pathological base of unfavorable blood pressure regulation is attributable to increased levels of angiotensin II and aldosterone, which partly result from adipose tissue dysfunction [20]. Consistent with the literature, we also support the fact of adipose-cardiovascular interaction established in our previous findings, which proved lifestyle-induced overactivation of RAAS in *SHRSP* rats [9]. Here, we can underpin that dysfunctional adipose tissue can be associated with an altered adipokine profile. In light of the fact that adipose tissue is an extremely active endocrine organ, the slightest change in the biologically active adipokines may contribute to increased oxidative stress and inflammation, which can develop or aggravate cardiometabolic abnormalities. As our findings show, lifestyle intervention changed the adipokine pattern, since 12 weeks of high-fat diet resulted in opposite changes in leptin-adiponectin ratio compared to *SHRSP*/CTRL rats. In the group of adipokines, leptin hormone serves as an indicator of adipose tissue changes and obesity. Leptin is produced primarily by adipose tissue, but a relatively low concentration is secreted into circulation from other tissues, such as skeletal muscle, brain, or stomach. After binding to the receptors, leptin exerts its effects in the central nervous system and peripheral organs as well [21]. 

Among experimental animal models for cardio-metabolic studies, both SHR and *SHRSP* phenotypes are characterized by the absence of excessive fat accumulation. However, although *SHRSP* rats are less prone to obesity in the literal sense compared to *Sprague-Dawley (SD)* or *Wistar* rats, lifestyle interventions have an impact on their metabolism. Although the mechanism of adiposity-related processes is well-described in obesity-prone rats, the exact mechanisms in SHR or *SHRSP* rats are not fully elucidated and need to be supported by more studies. Recently, Borghi et al. examined the influence of hypertensive environment on adipose tissue remodeling and their results suggest that low adiposity in SHR rats can be correlated with elevated thyrotropin-releasing hormone (TRH) activity and hypertension. They found that the leptin level is significantly decreased in SHR rats compared to the matched *Wistar-kyoto* control rats; however, serum adiponectin values were elevated in SHR rats [22]. In another study, Cao et al. described that lifestyle change (15 weeks of high-fat dietary intake) resulted in a significant increase in plasma levels of inflammatory cytokines, whereas adiponectin levels decreased compared to untreated SHR rats; however, they did not examine the adipokine profile of adipose tissue [23]. Similar to these results, we found here that 12 weeks of high-fat diet induced a significant increase in serum leptin concentration compared to CTRL rats. In contrast, adiponectin concentration measured from both adipose tissue and serum was significantly decreased in HFD rats compared to CTRL animals. Adiponectin is another adipokine expressed almost exclusively by adipose tissue. Additionally, pleiotropic effect is attributed to adiponectin, since it exerts not only cardioprotective functions, but also protective against insulin resistance and inflammatory processes [24]. In a comprehensive review, Zhao et al. showed that adiponectin secretion is determined by the quality of adipose tissue, rather than the amount of adipose tissue [14]. It is documented that healthy individuals display higher levels of circulating adiponectin compared to metabolically abnormal (unhealthy) individuals [25]. In accordance with our previous findings [9], adiponectin concentration changes measured in both adipose tissue and serum verify the fact that adiponectin possesses cardioprotective effects in individuals/rats with obesity-related or metabolically altered complications such as hypertension [26]. In order to support the fact that lifestyle changes exert direct effects on the interplay of adipose tissue and vasculature, further bioactive products, namely omentin and chemerin, were determined. Omentin represents a novel adipocytokine that exists abundantly in visceral fat tissue and serum as well. Omentin plays an important role in maintaining metabolism and cytoprotective mechanisms. Its anti-inflammatory and antiatherosclerotic effects are also described; thus, omentin serves as a potential biomarker for adipose-cardiovascular crosstalk [27]. Although it is a novel biomarker, previous studies have shown that lifestyle factors (e.g., dietary intake and exercise) can modify omentin-mediated effects [28]. Similar to the changes in adiponectin changes, our results also demonstrated that a high-fat diet decreased the omentin concentration of the adipose tissue. In contrast, chemerin adipokine has an opposing effect. Whereas omentin and adiponectin concentrations exert anti-inflammatory and cardioprotective properties, chemerin positively correlates with the induction and progression of cardiovascular diseases. In a recent review study, Ferland et al. introduce the role of chemerin as a driver of hypertension [29]. Parallel lines of research show that the role of chemerin has significant impacts on inflammatory and metabolic signaling mechanisms. In our work, we found that 12 weeks of 40% fat-contained dietary intake caused a significant elevation in the chemerin concentration of serum samples. Despite the increase of chemerin and leptin in serum, we did not observe changes in the leptin-chemerin concentrations of adipose tissue. Hojna et al. shed new light on the quality of adiposity. They proved that SHR strain is a unique model of adiposity. In the case of these rats, white fat depots contain brown-like adipocytes, which are characterized by energy expenditure, making this strain protected against excessive obesity [30]. These quality changes and different locations of fat deposits may result in region-specific adipokine expression. 

**Figure 7:**
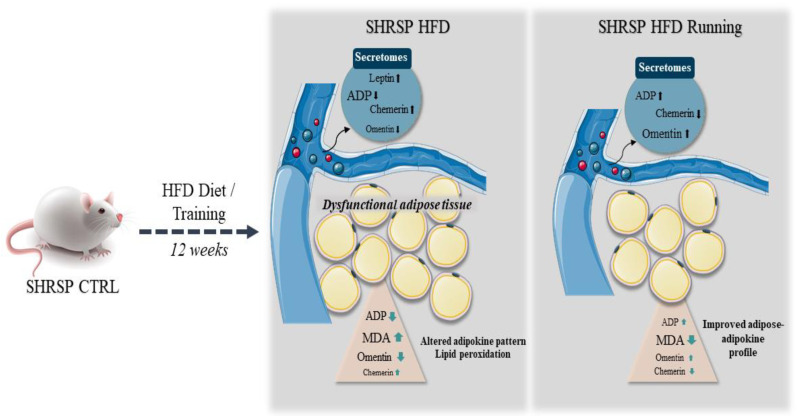
Changes in adipokine profile and lipid peroxidation at the end of the 12-week experimental protocol. ADP = adiponectin, CTRL = control, HFD = high-fat diet, MDA = malondialdehyde, *SHRSP*= spontaneously hypertensive stroke-prone rat.

Based on these alterations, preventive and therapeutic strategies have a significant role in managing the adipose tissue dysfunction and the consequent changes in the adipokine profile. One of the most important strategies to reduce the adverse effects is exercise training. It is well-established that exercise training, as a non-pharmacological treatment, can ameliorate oxidant/antioxidant homeostasis as well as reduce systemic and local inflammation in various diseases. In our experiment, *SHRSP* rats were used to examine the potential impact of exercise on adipose tissue and its secretomes. Since the *SHRSP* strain has severe hypertension, which is up-regulated by a high-fat diet, the type of physical exercise training is one of the critical points of the study [31]. In order to avoid the stressful environment and to take preventive/therapeutic advantages of the physical exercise, our training model was defined as voluntary wheel running exercise. In our earlier studies, we proved that voluntary wheel running exercise improved antioxidant status and decreased inflammatory parameters in different tissues. The protective mechanisms are also involved in the release of adipokines. We found that 12 weeks of wheel-running exercise ameliorated the HFD-induced adverse adipokine profile, while both adiponectin and omentin levels were increased in HFD/exercise groups. Previous studies underpin a direct relationship between increases in omentin and adiponectin levels and indicate that omentin regulation may be dependent on adiponectin; although, the expression of the adipokines is different in each adipose tissue deposit [32,33]. Similar to our findings, Castro et al. demonstrated that 12 weeks of aerobic exercise training increased adiponectin and omentin levels, whereas inflammatory parameters are decreased in a type 2 diabetes mellitus rat model [34]. Low-grade chronic inflammation is a key feature of oxidative stress, which contributes to cellular dysfunction and altered adipokine pattern, vice versa, as a result of ROS accumulation [6]. Oxidative stress can irreversibly damage macromolecules and disrupt redox signaling mechanisms. One of the main targets of ROS and the consequential oxidative stress is lipid content. Among the many different aldehydes which can be formed as secondary products during lipid peroxidation, elevated levels of malondialdehyde (MDA) serves as a biomarker for damage to adipose tissue. Obesity or high-fat diet-induced oxidative stress can be characterized by lipid peroxidation and elevated MDA level. In our study, 12 weeks of high-fat diet significantly increased the MDA level, whereas voluntary wheel-running exercise improved this pathological change. In our study, only inguinal adipose depot was examined; however, further measurements are needed in the future to obtain a deep insight into the interplay of lifestyle habits and adipokines. The main findings of the study are presented in Figure 7. 

## 5. Conclusions

Although obesity-related changes in adipokine levels are well-established, few research findings are available that examine the interplay of lifestyle changes and unfavorable cardiovascular conditions in light of adipose tissue function. Our results support the notion that energy-dense dietary intake contributes to adipose tissue dysfunction and adverse changes in an adipocyte-derived adipokine pattern. However, voluntary wheel-running exercise is a potential non-pharmacological strategy to ameliorate adipokine profile and to mitigate lipid peroxidation.

## Figures and Tables

**Table 1:** (**a**) Body weights at the start and at the end of the experimental protocol. Data are presented in b.w. gramm. The results are shown as the mean ± S.E.M. *n* = 6–8, *WKY* = *Wistar-kyoto* (control) rats, CTRL = standard chow, HFD = high-fat diet, R = running, *SHRSP* = *stroke-prone spontaneously hypertensive* rats. (**b**) Body weight changes during the experimental period (calculated from the differences between the individual initial and the individual final body weight of the animals). The results are shown as the mean ± S.E.M. *n* = 6–8, # *p* < 0.05: statistical significance between running vs. the respective non-running groups. *WKY* = *Wistar-kyoto* (control) rats, CTRL = standard chow, HFD = high-fat diet, R = running, *SHRSP = stroke-prone spontaneously hypertensive* rats.

		*WKY* CTRL	*SHRSP* CTRL	*SHRSP* CTRL + R	*SHRSP* HFD	*SHRSP* HFD + R
(a)	Body weight at the start of the experiment	157.25 ± 2.66	146.17 ± 4.5	156.50 ± 5.15	143.25 ± 5.94	192.25 ± 6.39
	Body weight at the end of the experiment	294.5 ± 1.93	258.83 ± 2.33	294.0 ± 2.98	303.63 ± 2.62	295.38 ± 4.14
(b)	Changes in the body weights	137.500 ± 44.863	112.667 ± 44.088	160.375 ± 5.322 #	103.125 ± 3.461	137.250 ± 4.109 #

**Table 2:** (**a**,**b**): Statistical table of the interactions between rat running and diet factors on adiponectin concentrations.

Source of Variation	*p* Value	
	(a)	(b)
**Interaction** **between Running × Diet**	0.305	0.066

**Table 3:** (**a**,**b**): Statistical table of the interactions between rat running and diet factors on leptin concentrations.

Source of Variation	*p* Value	
	(a)	(b)
**Interaction** **between Running × Diet**	0.836	0.680

**Table 4:** (**a**,**b**): Statistical table of the interactions between rat running and diet factors on omentin concentrations.

Source of Variation	*p* Value	
	(a)	(b)
**Interaction** **between Running × Diet**	0.751	0.290

**Table 5:** (**a**,**b**): Statistical table of the interactions between rat running and diet factors on chemerin concentrations.

Source of Variation	*p* Value	
	(a)	(b)
**Interaction** **between Running × Diet**	0.251	0.127

**Table 6:** Statistical table of the interactions between rat running and diet factors on MDA concentrations.

Source of Variation	*p* Value
**Interaction** **between Running × Diet**	0.390

## Data Availability

All data used to support the findings of this study are included within the article.
